# Supplementary material for: Temporal Trends in Acute Coronary Syndrome Mortality in Serbia in 2005–2019: An Age–Period–Cohort Analysis Using Data from the Serbian Acute Coronary Syndrome Registry (RAACS)
Source: Int J Environ Res Public Health. 2022 Nov 4;19(21):14457. doi: 10.3390/ijerph192114457 (PMC9659020; doi:10.3390/ijerph192114457)
Supplement: Supplementary file 1 [file ijerph-19-14457-s001.zip › Supplement File S1.pdf]

Calculation of reference age, reference period and reference cohort

Analyzed period: 2005-2019
Analyzed age groups: 20-90
Interval: 5-year intervals

| | | | Reference value |
|---|---|---|---|
| Reference Age | (Number of Age Groups + 1)/2 | (14+1)/2 = 7.5 | 7th age range is the reference age range |
| Reference Period | (Number of Periods + 1)/2 | (3+1)/2 = 2 | 2nd period group is the reference period range |
| Reference Cohort | Reference Period – Reference Age + Number of Age Groups | 2-7+14 = 19 | 19th cohort group is the reference cohort range |
| | | | |
| Period Point | (Lower Value + Upper Value +1)/2 | (2010+2014)/2 = 2012.5 | 2012 |
| Age Point | (Lower Value + Upper Value +1)/2 | (50+54)/2 = 52 | 52 |
| Cohort Point | Reference Period Point – Reference Age Point | 2012.5 – 52 = 1960.5 | 1960 |
