# Supplementary material for: Temporal Trends in Acute Coronary Syndrome Mortality in Serbia in 2005–2019: An Age–Period–Cohort Analysis Using Data from the Serbian Acute Coronary Syndrome Registry (RAACS)
Source: Int J Environ Res Public Health. 2022 Nov 4;19(21):14457. doi: 10.3390/ijerph192114457 (PMC9659020; doi:10.3390/ijerph192114457)
Supplement: Supplementary file 1 [file ijerph-19-14457-s001.zip › Supplement File S4.pdf]

The graph shows how mortality in terms of ASR-E per 100,000 for men and women, both, changed (decreased) based on number of performed PCIs per million inhabitants (primary PCIs).

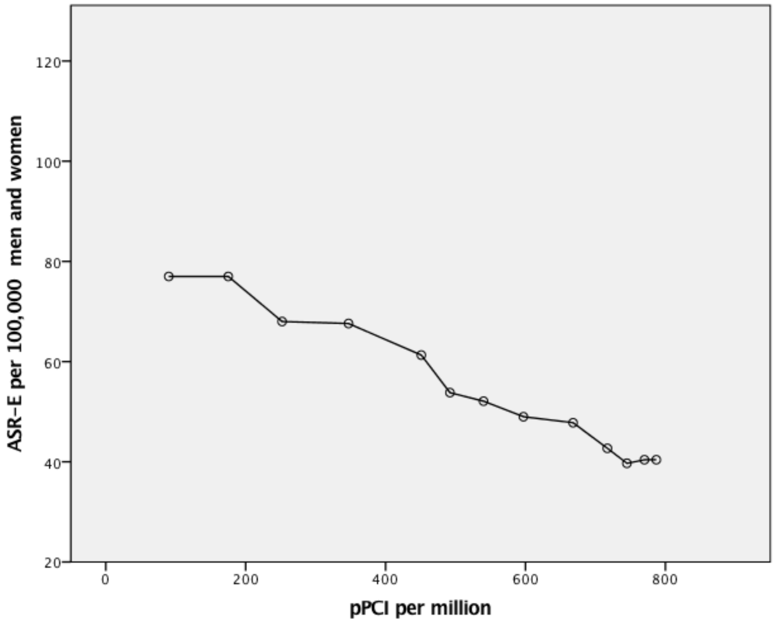

In men:

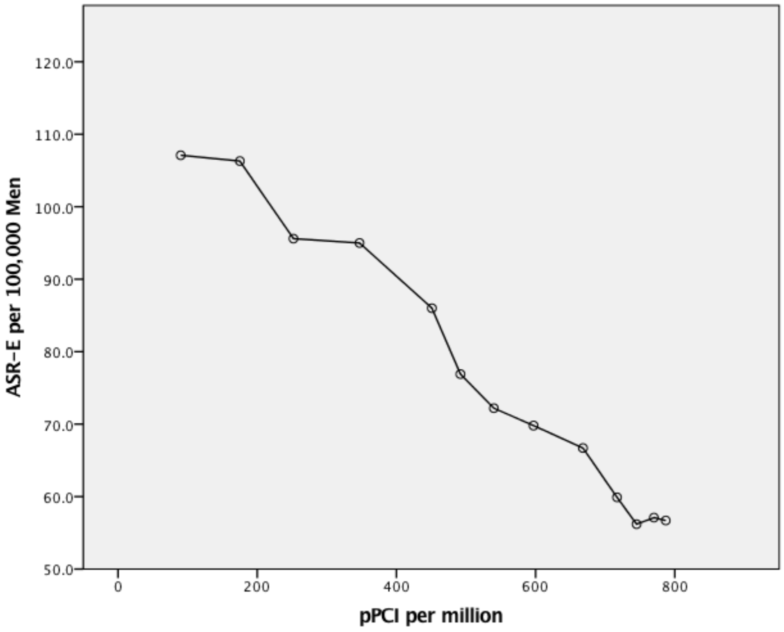

In women:

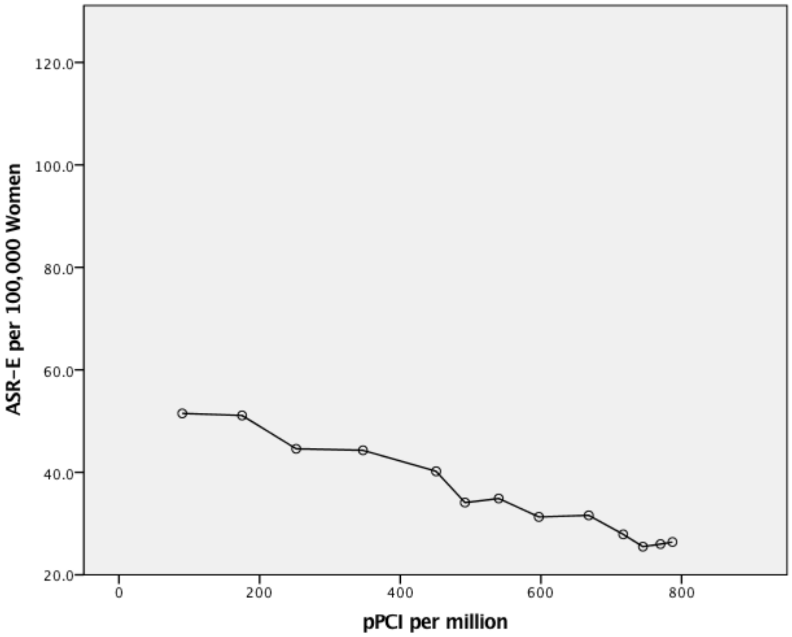

Frequency of use of reperfusion therapy in STEMI patients in first 90 minutes is a quality indicator since year 1994.
