# Supplementary figures and images for: Temporal Trends in Acute Coronary Syndrome Mortality in Serbia in 2005–2019: An Age–Period–Cohort Analysis Using Data from the Serbian Acute Coronary Syndrome Registry (RAACS)
Source: Int J Environ Res Public Health. 2022 Nov 4;19(21):14457. doi: 10.3390/ijerph192114457 (PMC9659020; doi:10.3390/ijerph192114457)

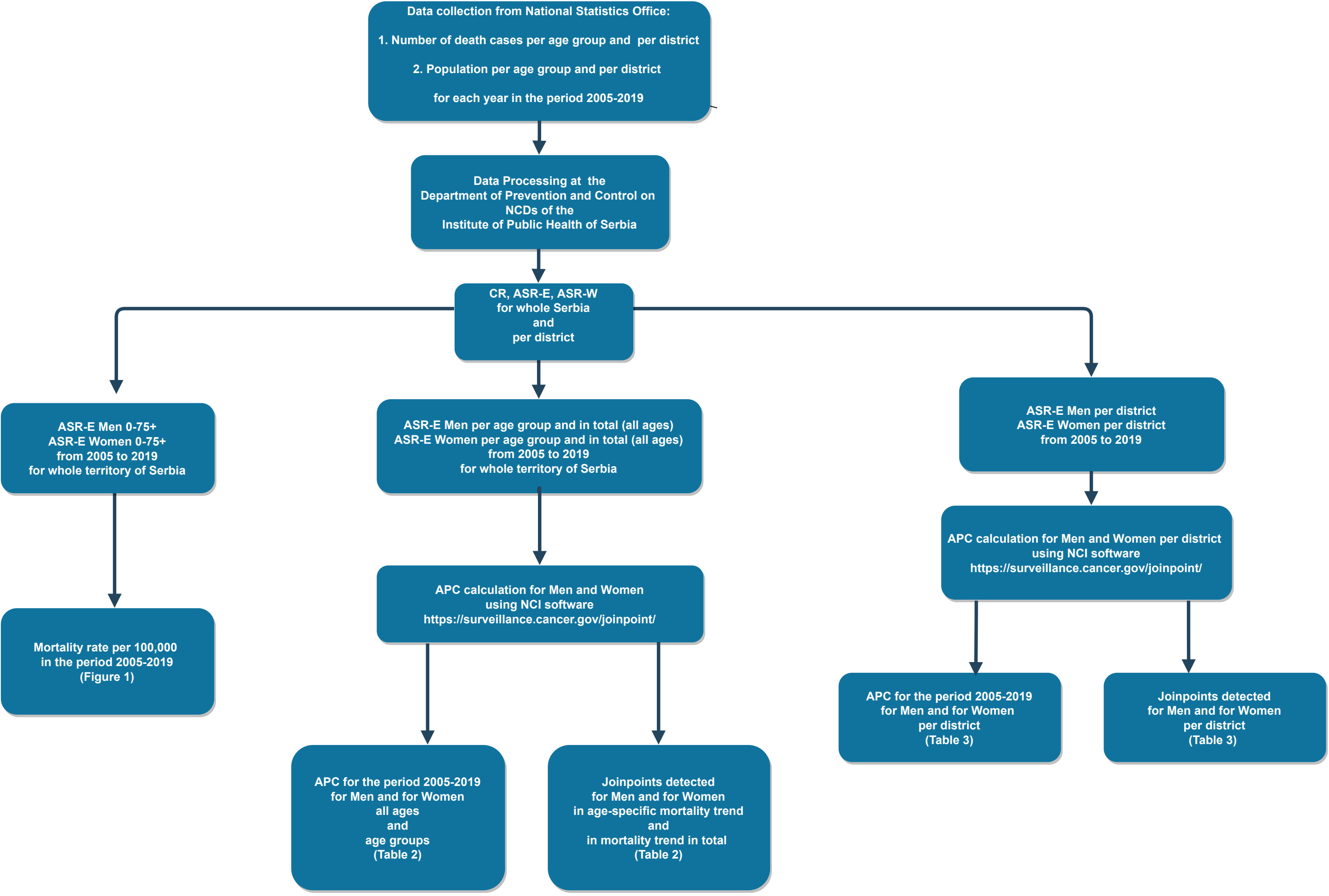

Supplement: Supplementary file 1 [file ijerph-19-14457-s001.zip › Supplement File S2.pdf]

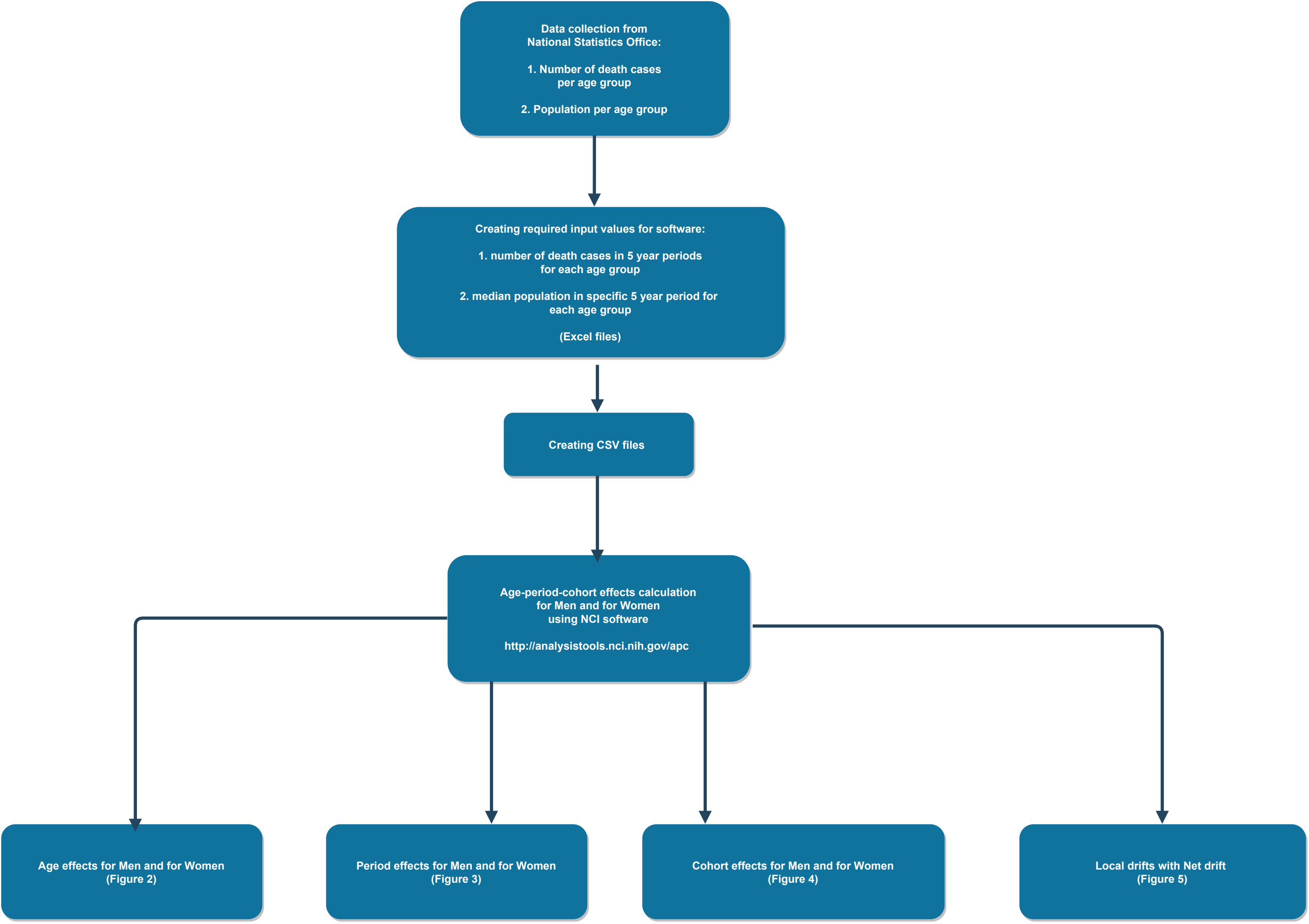

Supplement: Supplementary file 1 [file ijerph-19-14457-s001.zip › Supplement File S3.pdf]
